# Design and Optimization of SiON Ring Resonator-Based Biosensors for Aflatoxin M1 Detection

**DOI:** 10.3390/s150717300

**Published:** 2015-07-16

**Authors:** Romain Guider, Davide Gandolfi, Tatevik Chalyan, Laura Pasquardini, Alina Samusenko, Georg Pucker, Cecilia Pederzolli, Lorenzo Pavesi

**Affiliations:** 1 Nanoscience Laboratory, Department of Physics, University of Trento, Via Sommarive 14, Povo (TN) 38123, Italy; E-Mails: davide.gandolfi@unitn.it (D.G.); Tatevik.Chalyan@unitn.it (T.C.); samusenko@fbk.eu (A.S.); lorenzo.pavesi@unitn.it (L.P.); 2 LaBSSAH, Fondazione Bruno Kessler, Via Sommarive 18, Povo (TN) 38123, Italy; E-Mails: pasqua@fbk.eu (L.P.); pederzo@fbk.eu (C.P.); 3 Centre for Materials and Microsystems, Fondazione Bruno Kessler, Via Sommarive 18, Povo (TN) 38123, Italy; E-Mail: pucker@fbk.eu

**Keywords:** Whispering Gallery Mode, ring resonator, aflatoxin, waveguide, limit of detection, sensitivity, biosensor, label-free

## Abstract

In this article, we designed and studied silicon oxynitride (SiON) microring-based photonic structures for biosensing applications. We designed waveguides, directional couplers, and racetrack resonators in order to measure refractive index changes smaller than 10^−6^ refractive index units (RIU). We tested various samples with different SiON refractive indexes as well as the waveguide dimensions for selecting the sensor with the best performance. Propagation losses and bending losses have been measured on test structures, along with a complete characterization of the resonator’s performances. Sensitivities and limit of detection (LOD) were also measured using glucose-water solutions and compared with expected results from simulations. Finally, we functionalized the resonator and performed sensing experiments with Aflatoxin M1 (AFM1). We were able to detect the binding of aflatoxin for concentrations as low as 12.5 nm. The results open up the path for designing cost-effective biosensors for a fast and reliable sensitive analysis of AFM1 in milk.

## 1. Introduction

In the label-free biosensing approach, the target molecule (analyte) is selectively captured by a bio-recognition agent immobilized on the surface of the sensor. The trapped molecules form a layer with growing surface concentration that can be directly measured by quantifying the changes in the transmission spectra of the optical sensor [1].

It has been shown that Whispering Gallery Mode (WGM) resonators can be used as good label-free biosensors. They are made entirely with low-loss dielectrics and, for this reason, their resonances are much sharper and more resolved than Surface Plasmon Resonances (SPR) [2]. WGM resonators are appealing both for the very high quality that they can exhibit, as well as for the possibility of miniaturization down to a few tens of micrometers in diameter. Moreover, WGM are easily integrated with photonic waveguides to allow the realization of complex systems [3].

The possibility to realize small integrated and functional optical chips is particularly appealing for creating a biosensor. By miniaturizing the sensing sites, it is possible to limit the volumes of the samples or the chemicals involved in the detection protocol, reducing the costs and the time of every assay. The device itself can be cheaper because a high number of multiplexed sensors can be fit and run in parallel in a small area, as small as few square millimeters [4].

The goal of the European project Symphony [5] is the development of an integrated silicon-based photonic biosensor for the selective detection of Aflatoxin M1 (AFM1) in milk. This toxin, in fact, is a milk contaminant and potent carcinogen which is regulated by the European Commission (EC No. 1881/2006). Within this scope, we designed, realized, and characterized multiplexed label-free sensors based on silicon oxynitride (SiON) ring resonators integrated with a cheap Vertical Cavity Surface Emitting Laser (VCSEL) light source. The devices are also functionalized by immobilizing specific DNA aptamers with a silanization chemistry on their surface. These selective biosensors had to be able to distinguish small changes in the refractive index of bulk solutions flowing over their surface, as well as the formation of fractions of a monolayer of captured toxins.

This paper describes the characterization and optimization of the sensor. In the second section, we report on the design characteristics and on the simulated expected performances. In the third section, we report on the characterization of the test structures that were realized to optimize the design. Finally, we conclude our analysis by evaluating the sensing performances of our sensor and we prove the selective sensing of AFM1 in diluted buffer solutions.

## 2. Design and Simulation 

The photonic device proposed in this article is based on a low-cost, silicon (Si)-based, fully integrated optical sensor to detect the presence of aflatoxin in milk. As the absorption of water is three orders of magnitude higher in the infrared range than in the visible/near-infrared range, the operation wavelength of the sensor was chosen to be 850 nm. This choice entails other significant advantages since it allows using cheap photonic elements in our fully integrated system device, like Si-based photodiodes or VCSELs.

SiON films similar to SiN films, especially after thermal annealing, have a very large tensile stress which can easily result in fraction of the films. The film stress increases with the refractive index of the SiON. For this reason, we tested two different compositions of SiON to realize the optical components described here. The principle difference between them is the resulting refractive index (1.66 and 1.80) which, during deposition, is mainly controlled by the ratio of N_2_O to NH_3_ concentrations. To reduce the risk of fracture, definition of the optical components by reactive ion etching was performed prior to the thermal annealing. In this way the area of the wafer covered by highly stressed films is drastically reduced and relaxation of the residual stress is possible. For both compositions, inspection of the final structures performed with both optical and electron microscopy gave no evidence for fracture due to film stress.

The composition affects the film stress and the material losses. In addition, the two types of waveguides have a different refractive index contrast between waveguide and oxide cladding and, therefore, also have a different confinement of the optical modes. Use of waveguides with increasing refractive index allows for the realization of resonators with higher quality factors due to the smaller radiative losses. In Table 1, the description of all the processed wafers, in terms of thickness and refractive indices, are given.

**Table 1:** Wafer description.

Wafer Name	SiON Refractive Index	Deposited Thickness (nm)	Expected Annealing Shrinkage (%)	After Annealing Estimated SiON Thickness (nm)
L2	1.66	410	15	349
L5	1.8	240	10	216

### 2.1. Waveguide and Directional Coupler

In order to obtain a single mode waveguide, we simulated, using COMSOL Multiphysics software [6], the losses of SiON waveguides with widths varying between 900 nm and 1.4 µm. As initial parameters for the simulations, we chose a SiON waveguide thickness of 300 nm, a buried SiO_2_ (BOX) thickness of 3 µm, and a top cladding TEOS layer of 300 nm. In order to have a single mode waveguide at λ = 850 nm, we observed that the waveguide width should be narrower than 1.2 µm. Alternately, a small width increases the propagation losses of the waveguide. Therefore, we decided to set the values for the waveguide width at 900 and 1000 nm.

In order to perform multi-analyte sensing measurements, we needed to measure several sensors simultaneously, and thus we decided to use a directional coupler to split the input light in our device [7]. Providing a sufficiently long coupling zone, such a device does not need a high lithography resolution, as for the case of Y-junctions, and allows the use of a large gap distance between waveguides (in our case, from 400 to 600 nm). The analysis and design of such a concept was achieved using the coupled mode theory, where we analyze the transfer of power between two parallel identical waveguides. Using such formalism, we write the coupling length *L_cp_*, which represents the length where the maximum power is transfered from one waveguide to the other one, as a function of the wavelength λ and of the effective index difference between even (*n_eff_^e^*) and odd (*n_eff_^o^*) eigenmodes:

(1) $$L_{cp}\;=\;\lambda /[2\;\times \;({n_{eff}}^{e}\;-\;{n_{eff}}^{o})]$$

Simulations were performed for both waveguide widths and gaps between the two waveguides of 400, 500, and 600 nm, respectively. By analyzing the effects of fabrication tolerances on the coupling length, we verified that the more robust splitter configuration, for both waveguide widths, was obtained using a gap of 600 nm.

To conclude our analysis on directional couplers, we performed a complementary study (using the Finite Element Method) with the aim of verifying the coupling length of the directional coupler and fine-tuned the design parameters previously found. According to these calculations, and in order to test their accuracy in the experimental realization, we designed and characterized several test structures with coupling lengths varying from 65 to 90 µm for waveguide widths of 1000 nm, and from 50 to 75 µm for the width of 900 nm. Within this range of values, we expected to identify the correct coupling length in order to have a perfect low-loss 50/50 splitter.

### 2.2. Bending Radius and Ring Resonator Structures

The sensing device that we propose in this article is based on ring resonators. In order to design an efficient photonic layout, we initially investigated via finite element simulations, the bending losses of curved waveguides due to mode radiation. The study has been efficiently obtained in an axisymmetric 2.5D mode analysis that implemented a perfectly matched layer to account for the radiation losses [8,9]. According to our calculations, the waveguides show negligible radiation losses for bending radii higher than 100 µm. For shorter radii, the losses increase quickly, exceeding the value of 1 dB/cm before 60 µm, practically inhibiting the use of shorter radii. Once again, in order to confirm these calculations, we designed and characterized several test structures with multiple bending radii of 100, 75, 50, and 25 µm, both for waveguide widths of 0.9 μm and 1.0 μm.

Since higher refractive indexes allow for higher confinements, the ring thicknesses have to be optimized depending on the core material. We simulated rings with width w = 1 μm and radius R = 100 μm, while sweeping the ring thickness, and studied the influence on both quality factors and bulk sensitivity as in [9]. We considered core refractive indexes of both 1.8 and 1.66 for comparison. The refractive index in the top cladding is 1.33, assuming a sensor immersed in water, while the refractive index of the substrate is 1.45, corresponding to the silica.

**Figure 1:**
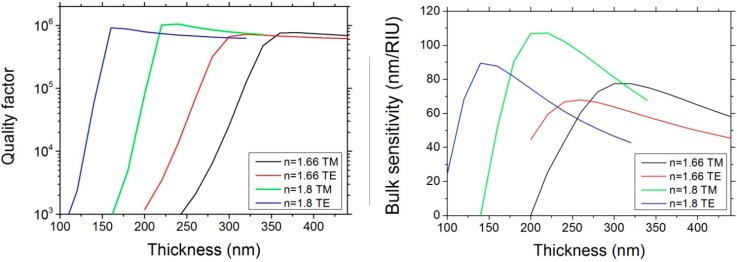
(**Left**) Quality factors in function of the waveguide thickness simulated for n_SiON_ = 1.66 and 1.8 for both transverse electric (TE) and transverse magnetic (TM) polarizations; (**Right**) Bulk sensitivity in function of the waveguide thickness simulated for n_SiON_ = 1.66 and 1.8 for both TE and TM polarizations.

While reducing the thickness, the mode confinement is reduced and the light-analyte interaction increases, thus enhancing the sensitivity. As represented in Figure 1, after a critical level, the mode starts to leak in the substrate, reducing the sensitivity in the upper cladding.

To couple the light to the ring resonator structures, we used the directional coupler theory, which is explained above. This geometry corresponds to the so-called racetrack resonator. To create such a structure, we first estimated the coupling coefficient between the waveguide and the resonator. The losses inside the resonator *α_r_* are related to the intrinsic quality factor through the following formula [10]:

(2) $$\alpha_{r}\;=\;(2\pi \;\times \;n_{g})/(\;\lambda \;\times \;O_{r})$$

where *n_g_* is the group index and *Q_r_* is the intrinsic quality factor of the resonator, which is twice the one measured in the critical coupling regime, *Q_tot_*. Since the fabrication process is in continuous optimization, the precise value of *α_r_* was not known. Based on literature and previous characterizations, we estimated *α_r_* to be in the range of 10^−2^ cm^−1^ (~0.1 dB/cm) [11] and 1 cm^−1^. From these values, we calculated the expected *Q_tot_* and the coupling coefficient, *k,* necessary to achieve a critical coupling [12]. Using the value of the coupling coefficient, it is finally possible to calculate the coupling length needed for a fixed gap to obtain such transfer of power.

**Figure 2:**
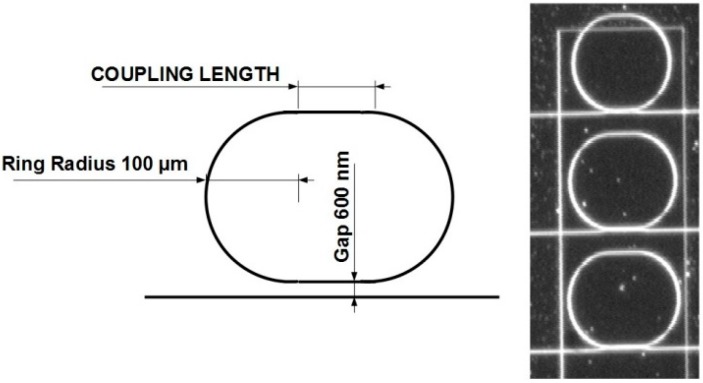
Sketch and microscope picture of the ring resonators sample. In the microscope image, we can clearly observe the etching windows around the resonators that allow the functionalization of the sensors.

According to our calculations, we designed several racetrack structures with coupling lengths varying between 0 and 64 µm, with a gap of 600 nm. Such a range of values should allow us to identify the optimal coupling length depending on the quality factor of the resonators. A sketch and microscope image of the sample is presented in Figure 2.

## 3. Experimental Characterization

To characterize the samples described above, we used a standard waveguide setup with two tapered fibers for the visible wavelengths placed on multiaxis translation stages. Six piezoelectric movements allowed for sub-micrometric alignment at the input and the output of the waveguides. The polarization is controlled using a two-paddle polarization controller. An optical microscope coupled to a visible/IR camera was used for alignment and imaging. For the detection part, we used a Si transimpedance amplified photodetector. Finally, as light source, we used a ULM850-B2-PL VCSELs from Philips Technologie GmbH U-L-M Photonics connected to a single mode visible fiber.

### 3.1. Bending and Propagation Losses 

As explained in section 2, to estimate the bending losses of the samples, we characterized several waveguides with multiple curves (from 20 to 40) with different radii (from 100 µm until 5 µm) and compare the intensity at their output with the one of a single waveguide of the same length and without any curve. 

**Figure 3:**
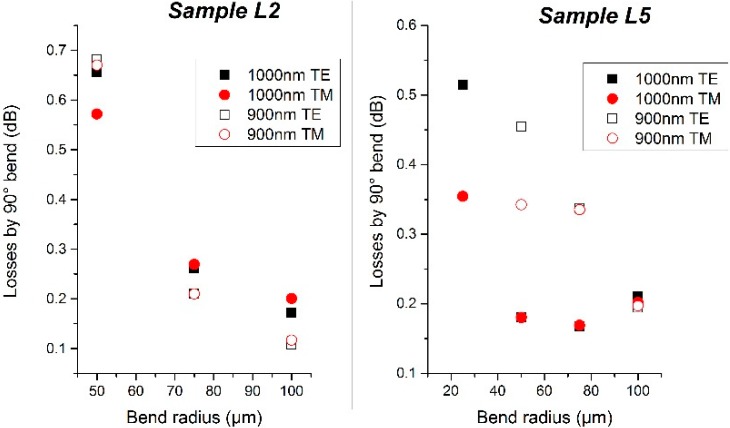
(**Left**) Bending losses of sample L2; (**Right**) Bending losses of sample L5.

The results of the bending losses for L2 and L5 samples are presented in Figure 3. Concerning the L2 wafer (n_SiON_ = 1.66), fairly high losses were found for waveguides of 1000 nm width and a bending radius of 100 µm (0.17 dB/90° bend), but the fact that lower losses were found for 900 nm width (0.1 dB/90° bend) leads us to think that this could be due to a problem in the coupling to the facet of the waveguide. For shorter radii, the losses increase exponentially. In the case of the L5 wafer (n_SiON_ = 1.8), bending losses below 0.2 dB/90° bend were found for radii spanning from 100 µm to 50 µm for waveguides of 1000 nm width. Concerning the 900 nm width waveguide, losses are in the range of 0.2 dB/90° bend, while they increase drastically for shorter radii. Such measurements confirm the low losses for 100 µm radius structures, and thus the possibility of high performances sensors based on ring resonators. 

In order to calculate the propagation losses of such waveguides, a simple method is to create “serpentines” waveguides. By using the same number of curves for each waveguide and changing the distance between each curve, it is possible to have waveguides with identical dimensions and different lengths on the same sample. By measuring the intensity at the output of the waveguides for the same input power, it is then possible to determine the propagation losses of the waveguide. It is important to note that in the calculation of the propagation losses, we had to subtract the losses due to the 100 µm radius curve of the “serpentine” design for both L2 and L5 wafers. Using this technique, we defined waveguides with total lengths of 5, 10, 20, and 30 mm. 

**Figure 4:**
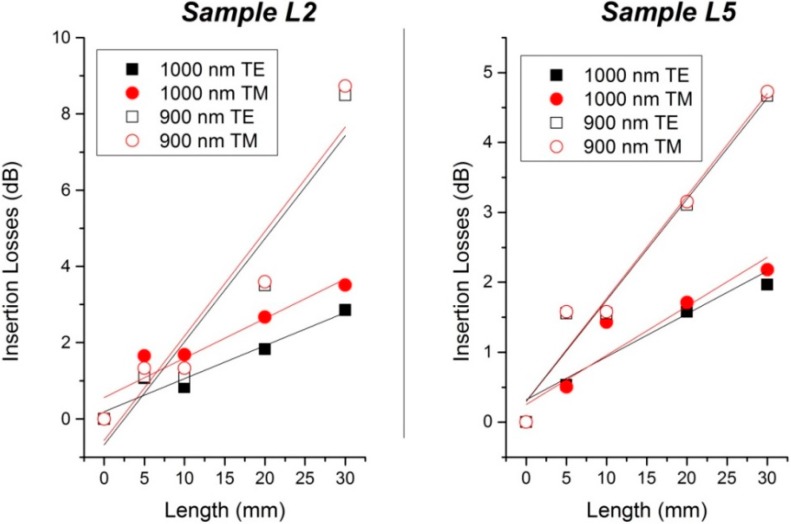
(**Left**) Propagation losses of sample L2; (**Right**) Propagation losses of sample L5.

The results of the propagation losses are presented in Figure 4 and summarized in Table 2.

**Table 2:** Propagation losses of L2 and L5 samples.

Sample	Waveguide Width (nm)	Propagation Losses TE (dB/cm)	Propagation Losses TM (dB/cm)
L2	900 nm	2.70	2.70
	1000 nm	0.87	1.00
L5	900 nm	2.00	2.00
	1000 nm	0.61	0.70

### 3.2. Directional Coupler Characterization

**Figure 5:**
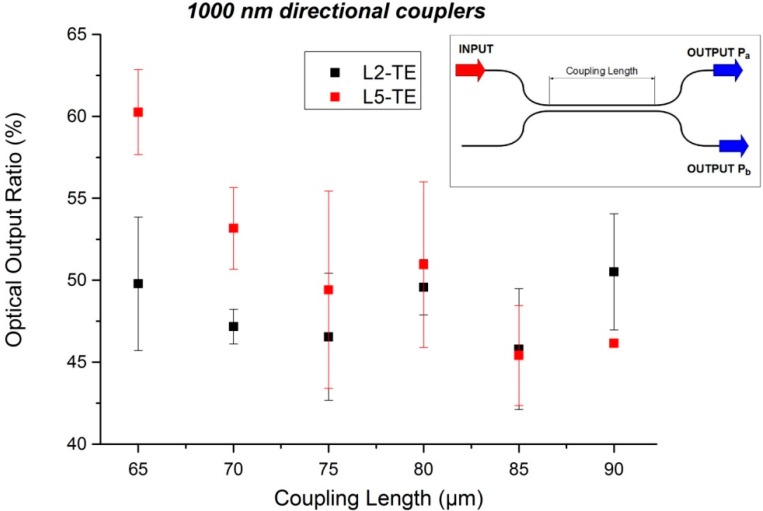
Optical characterization of the directional coupler for L2 and L5 samples. The optical output ratio represents the optical power at the output waveguide (P_a_) *versus* the sum of the optical power of both output waveguides (P_a_ + P_b_). (**inset**) Scheme of a directional coupler.

As the previous results were really promising concerning the 1000 nm waveguide, we decided to focus the rest of our analysis on waveguides of such dimensions. Concerning the directional coupler measurements, we injected the light into one arm for each directional coupler and check the optical power at the outputs of the same waveguide (P_a_) and of the parallel one (P_b_). A scheme of the directional coupler device is presented in the inset of Figure 5. The ratio between output power P_a_ and the sum of output powers (P_a_ + P_b_) is represented in Figure 5 for wafers L2 and L5.

We observed a balanced splitting ratio for wafers L2 at a coupling length of around 77.5 µm in TE polarization. Concerning 1.8 refractive index SiON wafers, wafer L5 is close to a good 50 × 50 splitting ratio around a 75 µm coupling length.

### 3.3. Ring Resonator Characterization 

**Figure 6:**
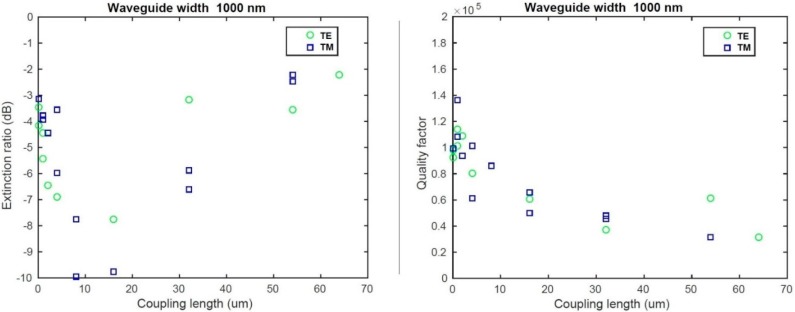
(**Left**) Extinction ratio analysis for the L2 sample; (**Right**) Quality factor analysis for the L2 sample.

**Figure 7:**
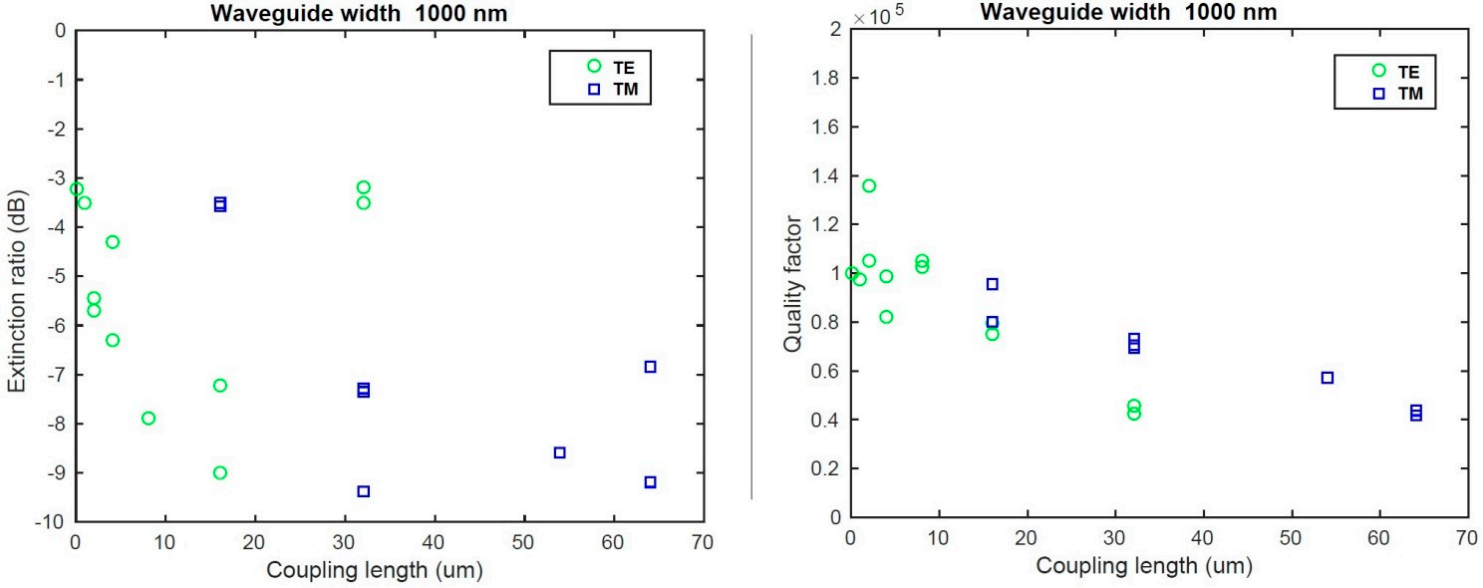
(**Left**) Extinction ratio analysis for the L5 sample; (**Right**) Quality factor analysis for the L5 sample.

According to our simulations, we analyzed the quality factor of the ring resonators for both L2 and L5 wafers for the waveguide width of 1000 nm. We measured resonators covered with an oxide cladding and with different coupling lengths in order to find the critical coupling regime and thus the intrinsic quality factor of the resonator. Results are presented in Figure 6 for the L2 wafer and in Figure 7 for the L5 wafer.

Concerning the L2 wafer, we observed critical coupling for a coupling length of 15 ± 5 µm for both polarizations. At such a coupling length, we measured a quality factor *Q_tot_* of 6 × 10^4^ (±10^4^) for 1000 nm width in TE polarization. Such values lead us to an intrinsic quality factor of 1.2 × 10^5^ (±2 × 10^4^). In TM polarization, an intrinsic quality factor of 1.4 × 10^5^ (±2 × 10^4^) was measured for 1000 nm width.

For the L5 wafer, we observed critical coupling for the 15 ± 5 µm coupling length in TE polarization and 40 ± 5 µm in TM polarization. At such a coupling length, we measured a quality factor *Q_tot_* of 7.5 × 10^4^ (±1 × 10^4^) for 1000 nm width in TE polarization. Such values lead us to an intrinsic quality factor of 1.3 × 10^5^ (±1 × 10^5^). In TM polarization, an intrinsic quality factor of 1.3 × 10^5^ (±1 × 10^5^) was measured.

## 4. Sensitivity, Sensing Measurements, and Discussion

### 4.1. Sensitivity and Limit of Detection 

**Figure 8:**
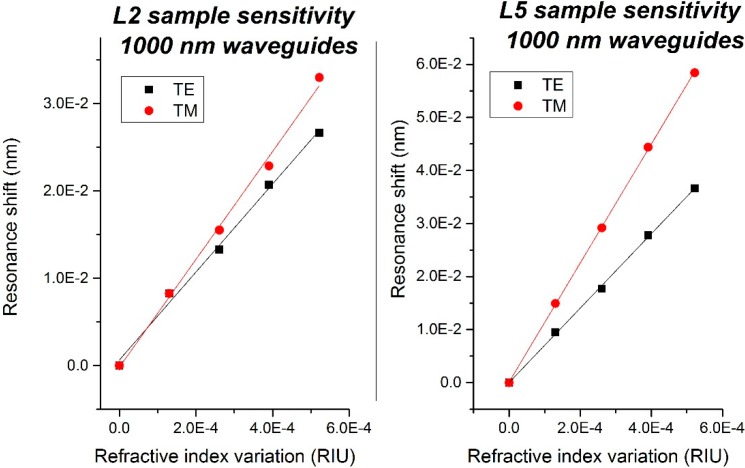
(**Left**) Sensitivity of the L2 sample; (**Right**) Sensitivity of the L5 sample.

According to the high quality factors measured on covered resonators, we also designed and measured uncovered rings fabricated on the same wafers. These resonators are used as sensors, as the removal of the top cladding layer allows their functionalization. In order to characterize them, we measured their sensitivity as S = Δλ/Δn. To determine the sensitivity of the ring resonator sensors, we monitored the shift of the WGM resonances while exposing the sensor to several glucose*-*water solutions of different concentrations. More details on this experiment are available in [13]. Figure 8 represents the bulk sensitivity of the L2 and L5 wafers as a function of the polarization.

We measured a homogeneous sensitivity of 60 nm/RIU in the case of the L2 wafer, and a higher value of 112 nm/RIU in the case of the L5 wafer. 

The limit of detection (LOD) of our sensors is defined as the minimum input quantity that can be distinguished with more than 99% fidelity [13], and it is determined as the ratio between three times the output uncertainty and the sensitivity of our sensor. In our case, we measured a LOD of 1.6 × 10^−6^ RIU on sensors from wafer L5, and 3 × 10^−6^ RIU with L2 samples. 

### 4.2. Aflatoxin-Sensing Measurements

The final goal of our sensors is the selective sensing of Aflatoxin M1 in buffered solutions. To test the feasibility and the limits of such detection, we functionalized the samples by using a wet silanization process [13] using a DNA aptamer that recognizes AFM1 with high affinity and specificity [14,15]*.* The wet silanization process is followed by a deposition and immobilization of the amino-modified anti-aflatoxin DNA aptamer. As a final process, a passivation step with 1mM ethanolamine in the same buffer for 30 min completed the procedure. 

**Figure 9:**
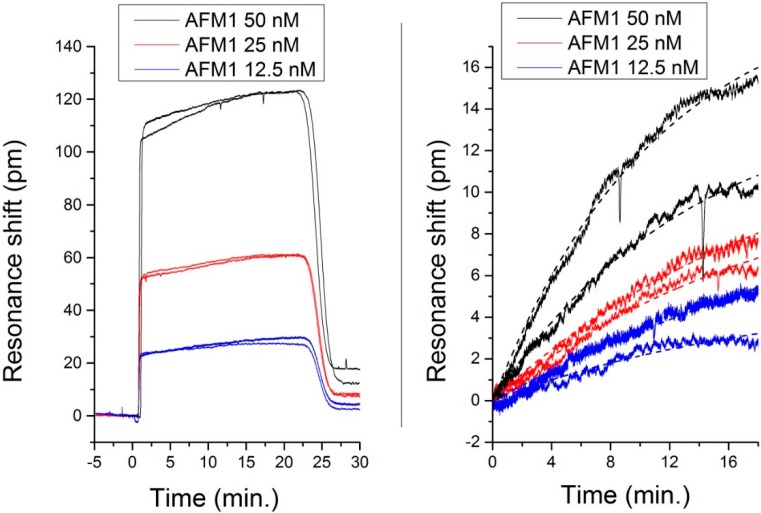
(**Left**) Sensorgram recorded using a sample from the L2 wafer in TM polarization. The high step-like response is due to the refractive index mismatch produced by the small content of Dimethyl sulfoxide (DMSO) in the solution. This solvent is needed to dissolve the AFM1 in the buffer solution; (**Right**) Specific binding sensorgrams obtained from the curves in (**Left**) by subtracting the bulk shift induced by the DMSO content. The dashed curves are exponential fittings for the evaluation of the rate constants and of the initial slopes [4].

To perform our sensing experiments, we flew several solutions containing different aflatoxin concentrations over the sensor devices. More details on the experimental process are available in [13]. Figure 9 represents the sensorgrams obtained for the L2 sample in TM polarization. Between each measurement, we performed an injection of glycine solution (100 mM glycine-HCl, pH 2) in order to regenerate the aptamers on the surface of the sensors.

We can appreciate the clear dependence of these signals on the toxin concentration. Another important observation on these measurements is the fact that we could use the same sensor for many injections (nine AFM1 injections and nine glycine injections), demonstrating that the use of glycine can effectively regenerate the surface of these sensors. It is important to note that these measurements were effectuated in a temperature-controlled laboratory in order to keep the ambient temperature of the setup constant. The VCSEL used for the measurements was also connected to a temperature controller with a temperature resolution of 0.01 °C. We noticed a perfect stability of the signal of the resonators over minutes, with a drift below 0.1 pm/min. Moreover, in the final sensor design, we plan to split the input light into four different ring resonators, and to functionalize them with different aptamers. The difference in the response between the differently functionalized sensors will give us information about the presence of aflatoxin in the solution. Using such a concept, any thermal change in the sample or solution will be compensated for.

Finally, considering a real application of the functionalized sensor, we have to consider that this work is in the framework of the Symphony project and a pre-purification module is under development. The solution that will be driven over the functionalized sensor will therefore be purified and cleaned from proteins and contaminants.

## 5. Conclusions

In this article, we designed and tested SiON biosensors based on microring resonators. Simulations were effectuated in order to optimize the structures' performances and different batches of samples were characterized. On optimized samples, we achieved a high sensitivity (S = 112 nm/RIU) and low LOD (LOD = 1.6 × 10^−6^ RIU). We finally performed sensing measurements on functionalized sensors. We observed selective binding at different aflatoxin concentrations, as well as a very good regeneration efficacy when using glycine. 

This study will allow the creation of a fast and reliable sensor, without the need of a high lithography resolution, based on photonic ring resonators with high performances, with applications in the areas of environmental monitoring, agrofood, clinical medicine, or healthcare. Such a study could open the path to the realization of a complete lab-on-chip Si-based system with a source, sensor, and detector integrated in a unique photonic device.
